# Overexpression of a Xylem-Dominant Expressing BTB Gene, *PtrBTB82*, Influences Cambial Activity and SCW Synthesis in *Populus trichocarpa*

**DOI:** 10.3390/plants15010068

**Published:** 2025-12-25

**Authors:** Siran Zhu, Hongtao Yao, Jiayi Liu, Xiao Zhao, Jiyao Cheng, Chong Wang, Wenjing Xu, Chunming Li, Yuxiang Cheng

**Affiliations:** 1 State Key Laboratory of Tree Genetics and Breeding, Northeast Forestry University, Harbin 150040, China; imabcab@163.com (S.Z.); hongtaoyao@nefu.edu.cn (H.Y.); 15245371802@163.com (J.L.); chengjiyao1993@163.com (J.C.); nefuwangchong@126.com (C.W.); xuwenjinglucky@163.com (W.X.); 2 Qiqihar Branch of Heilongjiang Academy of Forestry, Qiqihar 161005, China; zx98111309@163.com

**Keywords:** cambial activity, SCW synthesis, *BTB/POZ* gene, overexpression, *Populus trichocarpa*

## Abstract

The BTB/POZ protein family is widely distributed across the biological kingdom, and its various subfamilies perform diverse physiological functions, including regulating plant growth and development, defending against pathogen invasion, participating in metabolic regulation, and responding to abiotic stresses. However, the functional roles of BTB genes in wood formation remain largely unknown. In this study, a total of 103 BTB genes were identified in *Populus trichocarpa*. Expression pattern analysis and β-glucuronidase (GUS) staining revealed that *PtrBTB82* was predominantly expressed in the xylem. Overexpression of *PtrBTB82* in *P. trichocarpa* significantly reduced cambial activity, resulted in a narrower xylem, and altered the chemical composition of the secondary cell wall, suggesting that *PtrBTB82* plays the roles in wood formation. Quantitative real-time PCR (RT-qPCR) analysis showed that overexpression of *PtrBTB82* suppressed the expression of genes related to the WUSCHEL-related pathway and plant hormone signaling, which may underlie the reduced cambial activity and inhibited xylem development. Moreover, genes associated with lignin biosynthesis (*PtrPALs*, *PtrC4H1*, *Ptr4CL* and *PtrCAD1*) were upregulated, while secondary wall cellulose synthase genes (*PtrCESA7A/B* and *PtrCESA8A*) were markedly downregulated in the overexpression lines, likely contributing to the altered chemical composition of the wood. Collectively, this study provides new insights into the role of *PtrBTBs* in wood formation, thereby revealing the functional diversity of the *BTB* family in plants.

## 1. Introduction

Wood, a terrestrial huge biomass, is a valuable renewable resource for human life and industrial products including construction, papermaking, and bioenergy. Wood formation in trees is a complex process that begins with the proliferation of vascular cambium cells and their inward differentiation into xylem cells, followed by cell enlargement, secondary cell wall deposition, and programmed cell death, which eventually results in mature wood [1]. Given the huge biomass generated annually by the vascular cambium and the wide range of applications for wood, understanding the molecular mechanisms of wood formation, especially cambial activity and secondary cell wall (SCW) synthesis is of great significance.

Vascular cambium regulates secondary growth and produces wood in woody plants via hormone signaling pathways and multilayered transcriptional networks [2]. Auxin signaling is critical for maintaining cambial activity [3]. Studies have shown that auxin levels in the cambial zone, which are mediated by IAA (Aux/IAA transcriptional repressors), ARF (auxin response factor), and PIN (PIN-FORMED protein), have a direct influence on cell division and xylem differentiation [4,5,6]. Meanwhile, cytokinin stimulates cell proliferation via the AHK/ARR signaling cascade and acts synergistically with auxin to maintain cambial activity [7]. Gibberellins and brassinosteroids work alongside auxin to control cambial cell division and vessel differentiation [8,9]. In woody plants, vascular cambium has a more sophisticated regulatory network, with *WOX4* identified as a key regulator of cambial activity [10]. *WOX4* stimulates the persistent proliferation of cambial cells via the conserved *CLE41/44-PXY-WOX4* signaling pathway, which maintains cambial activity and secondary growth [11]. In addition, various transcription factors (TFs) including *VCM1/2*, *VCH2/2h*, *PtrHB4*, *MYB31*, and *PaC3H17-PaMYB199* have been demonstrated to influence cambial cell layers and wood structure in *Populus* [2]. The integrations of hormone signaling and intricate transcriptional networks fine-tune vascular cambium development, controlling not only cell division and differentiation patterns, but also having a significant influence on wood SCW synthesis.

During wood formation, xylem cells such as fibers and vessels undergo a central development to thicken their cell walls, i.e., forming SCW structure. The main components of the SCWs are cellulose, lignin, and hemicellulose. Cellulose in SCWs is synthesized by the cellulose synthase (CesA) 4/7/8 complex, which determines wall thickening and microfibril orientation [12]. Lignin is composed of three monolignols: p-hydroxyphenyl (H), guaiacyl (G), and syringyl (S) lignin. These monolignols are derived from phenylalanine, which is first converted into cinnamic acid or p-coumaric acid by phenylalanine ammonia-lyase (PAL). Subsequently, they undergo a series of reductions catalyzed by 4-coumarate-CoA ligase (4-CL), cinnamoyl-CoA reductase (CCR), and cinnamyl alcohol dehydrogenase (CAD). Hydroxylation is catalyzed by p-coumaroyl shikimate 3-hydroxylase (C3H), cinnamate 4-hydroxylase (C4H), and ferulate 5-hydroxylase (F5H). Caffeoyl-CoA O-methyltransferase (CCoAOMT) and caffeic acid O-methyltransferase (COMT) catalyze methylation reactions, leading to the formation of the three lignin monomers [13]. Hemicellulose, predominantly xylan, is synthesized by glycosyltransferase complexes such as *IRX9/10/14A*, and its modifications regulate interactions with cellulose to maintain wall architecture and mechanical performance [14,15]. The changes in the chemical composition strongly influence wood properties, for example, reduced cellulose leads to thinner walls and decreased mechanical strength [16,17,18]. Therefore, the variation in SCW components determines the structural function and application potential of wood.

Wood formation is tightly regulated by an intricate transcription regulatory network [19,20]. *NAC* (NAM, ATAF1/2, and CUC2) family TFs such as *SNDs* (secondary wall-associated NAC-Domains) and *VNDs* (Vascular-related NAC-Domains) are recognized as upstream master regulators, capable of modulating the expression of downstream transcription factors at multiple tiers, thereby controlling the transcription of secondary cell wall biosynthetic genes for cellulose, hemicellulose, and lignin [21]. R2R3-MYB family members, as downstream factors, act as transcriptional activators that directly regulate genes involved in cell wall component biosynthesis, thus playing pivotal roles in promoting SCW deposition and thickening [22]. In addition to the *NAC–MYB* cascade, other TF families also participate in the SCW synthesis. KNOX proteins such as KNAT7 act as negative regulators of SCW deposition and members of the *WRKY*, *bHLH*, and *LBD* TFs participate in the regulation of SCW formation via diverse and independent regulatory pathways [23,24,25,26]. Although these investigations have identified a variety of TFs involved in wood formation, additional TF members in this process need to be discovered.

BTB (Broad-complex, tramtrack, and bric-a-brac) proteins, also known as POZ (Pox virus and zinc) zinc finger domain proteins, were first identified in Drosophila melanogaster [27]. Conserved BTB/POZ domain mediates dimerization and interactions with diverse proteins, playing key roles in transcriptional regulation, cytoskeletal stability, and ion channel regulation [28,29]. According to the type of associated domains, BTB proteins are classified into several subfamilies, including BTB-BACK, BTB-Kelch, BTB-Arm, BTB-MATH, BTB-TAZ, BTB-ANK, and BTB-NPH3 [27]. In plants, they participate in the regulation of plant morphogenesis, secondary metabolism, hormone signal transduction, and stress responses [30,31,32,33,34,35,36]. For example, MATH-BTB is involved in ABA signaling and embryonic development [37,38]; BTB-TAZ responds to various hormonal and environmental cues [39]; and BTB-NPH3 mediates phototropic responses [40]. In addition, the number of BTB family members varies greatly among different plant species [41,42,43,44,45], reflecting their evolutionary diversity and functional specialization. However, little is known on functional description of *BTB* gene in wood formation.

In this study, we identified a *BTB* gene family in *Populus trichocarpa*, and *PtrBTB82* was substantially expressed in secondary xylem. Overexpression of *PtrBTB82* in transgenic poplar inhibited vascular cambial activity, thereby limiting secondary xylem growth. Furthermore, overexpression of *PtrBTB82* influenced SCW synthesis, causing changes in wood chemical composition. Our findings provide new insights into the involvement of plant BTB gene in wood formation.

## 2. Results

### 2.1. Identification and Characterization of PtrBTB Genes in P. trichocarpa

To identify the *BTB* genes in *P. trichocarpa*, we searched its genome database using the BTB/POZ conserved domain (PF00651, IPR000210, IPR011333, and IPR016073). The result identified 103 genes, each of which encodes a protein with the PF00651 domain. These genes were designated *PtrBTB1* to *PtrBTB103* based on their chromosomal positions (Table S1). A phylogenetic tree showed that the 103 PtrBTB proteins were divided into eight subtypes: NPH3, only-BTB, Ankyrin, MATH, BACK, Armadillo, TPR, and TAZ (Figure S1). Among these subtypes, the NPH3 subtype has the most members and the Armadillo subtype the fewest. We also used the MEME web tool to examine the distribution of preserved motifs and found eight different motifs. All PtrBTBs contain either a BTB motif or a BTB/POZ motif, and the motifs within each subtype are highly similar, but there are differences within subtypes (Figure S2A). Exon-intron gene structure analysis revealed that each subtype had the same number of exons and introns, but there were significant differences in gene structure between subtypes (Figure S2B).

**Figure 1:**
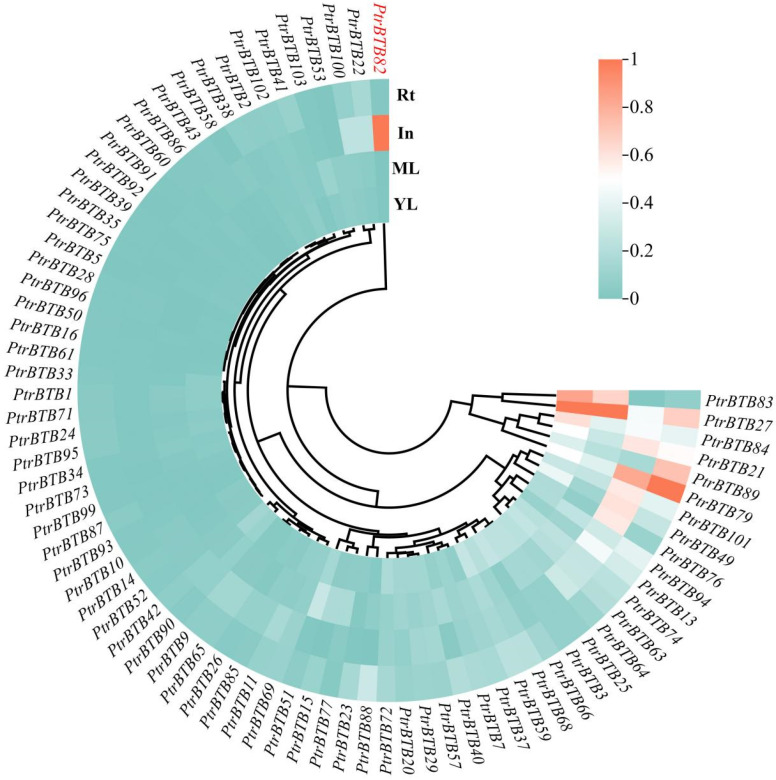
Hierarchical clustering of expression profiles of *PtrBTB* genes in different tissues. Microarray data were downloaded from the *Populus* eFP browser. The color scale at the right of the graph indicates the normalized expression levels, and the range is adjusted to 0–1. ML, mature leaf; YL, young leaf; RT, root; In, internode. The gene highlighted in red indicate that selected for this study.

We next used the poplar eFP Browser website (http://bar.utoronto.ca/efppop/cgi-bin/efpWeb.cgi; accessed on 14 March 2024) to assess the expression of *BTB* family genes in several poplar tissues, including root, internode, old leaf, and young leaf. Of the 103 *PtrBTBs*, 76 genes were recovered. After standardizing the expression data of these genes using the Min-Max Scaler, a heatmap was created to highlight their gene expression patterns in poplar tissues (Figure 1). *PtrBTB79* showed high expression levels in *P. trichocarpa* internodes and roots with rich secondary xylem tissues. Strikingly, *PtrBTB82* showed specifically high expression levels in internode tissue, indicating its potential significance in stem growth and development.

**Figure 2:**
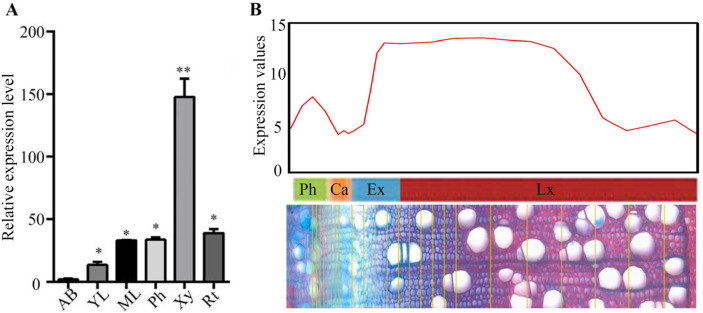
Transcriptional abundances of *PtrBTB82* in poplar various tissues. (**A**) Analysis of *PtrBTB82* gene expression levels in *P. trichocarpa* tissues using RT-qPCR. Relative expression levels were normalized using the geometric mean of *PtrActin2*. Differentially expressed genes were determined by |fold change| > 2, *p* < 0.05. Asterisks denote a significant difference between the apical buds and other tissues using Student′s *t*-test: * *p* < 0.05, ** *p* < 0.01. (**B**) The expression pattern of *PtrBTB82* across the stem tissue using AspWood RNAseq dataset. This diagram can be roughly divided into four parts, phloem (Ph), cambium zone (Ca), expanding xylem area (Ex), and lignified xylem area (Lx). In the table, each dashed line corresponds to the starting position of its respective region.

To further investigate the expression pattern of *PtrBTB82*, we collected total RNAs from *P. trichocarpa*’s apical buds, young leaves, mature leaves, phloem, xylem, and root of and used RT-qPCR to assess its transcription levels in different tissues. Among the tissues studied, xylem had the highest transcription levels of *PtrBTB82* gene (Figure 2A). Using the ASPWood RNA-seq database (http://aspwood.popgenie.org; accessed on 26 May 2024), we examined the transcription levels of *PtrBTB82* in various stem tissues, including phloem, cambium, expanding xylem, and lignified xylem. The result showed that *PtrBTB82* had the highest transcription levels in developing xylem (Figure 2B), implying its involvement in wood formation.

### 2.2. Promoter Activity of PtrBTB82 Gene in ProPtrBTB82::GUS Transgenic Populus

**Figure 3:**
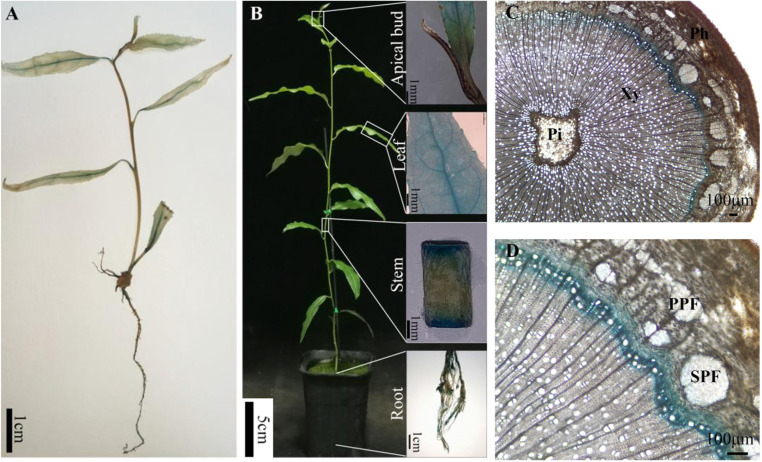
Histochemical GUS assays in various tissues of *ProPtrBTB82*::GUS transgenic *P. trichocarpa*. (**A**) One-month-old transgenic plantlets on woody plant medium. (**B**) Three-month-old *ProPtrBTB82*::GUS transgenic young trees in a greenhouse. (**C**) A cross-section of the 10th stem internode from three-month-old transgenic young trees. Pi, pith; Xy, xylem; Ph, phloem. (**D**) Magnified cross-section from (**C**). PPF, primary phloem fiber; SPF, secondary phloem fiber.

To study the promoter tissue expression pattern of *PtrBTB82* gene, the promoter region (1815 bp) was extracted, and a *ProPtrBTB82*::GUS binary expression vector was constructed. Following transformation into wild-type (WT) *P. trichocarpa*, we created *ProPtrBTB82*::GUS transgenic plants that expressed a GUS gene driven by the *PtrBTB82* promoter. GUS staining was applied to 1-month-old transgenic plantlets and 3-month-old transgenic young trees, respectively. GUS staining was visible in the leaf veins, petioles, and roots of one-month-old transgenic plantlets, but no GUS staining was detected in the apical buds or stem skins (Figure 3A). In three-month-old transgenic trees undergoing secondary xylem, GUS staining was mostly localized to leaf veins, stems, and roots, showing that *PtrBTB82* promoter tissue expression activity is limited to vascular tissues (Figure 3B). Further examination of stem cross-sections revealed that GUS activity was mostly concentrated in developing xylem fiber cells, cambium, and adjacent parenchyma cells, with no activity in xylem vessel cells or phloem fiber cells (Figure 3C,D). These data indicate that *PtrBTB82* is likely involved in xylem development.

### 2.3. Phenotypes of PtrBTB82-Overexpressing P. trichocarpa

**Figure 4:**
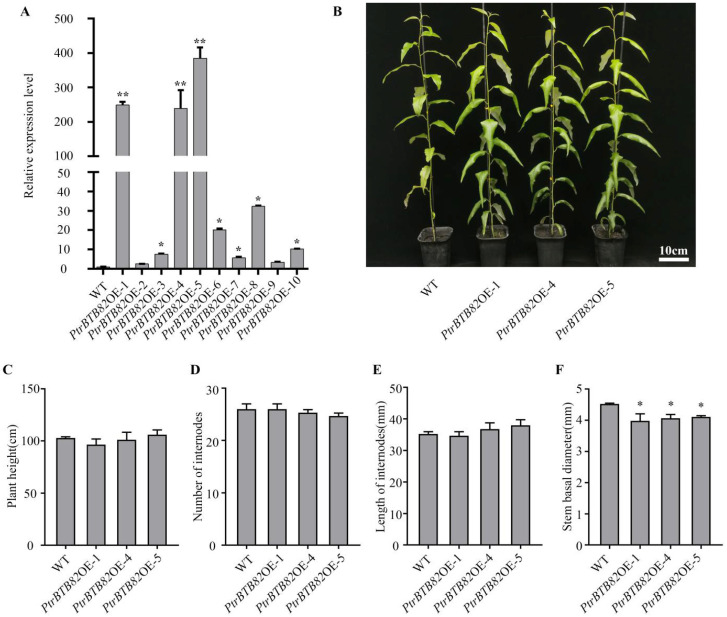
Phenotypes of *PtrBTB82* overexpressing plants. (**A**) RT-qPCR analysis of *PtrBTB82* transcription levels in wild-type (WT) and transgenic *P. trichocarpa*. A change of more than twofold was used to determine differential expression. (**B**) Morphology of WT and *PtrBTB82OE*-1/4/5 transgenic lines cultivated in a greenhouse for three months. (**C**–**F**) A statistical examination of plant height (**C**), internode number (**D**), internode length (**E**), and stem basal diameter (**F**) in WT and transgenic lines. The values are means ± SDs (n = 3). Asterisks indicate a significant difference from WT using the Student′s *t*-test (* *p* < 0.05; ** *p* < 0.01).

To explore the gain-of-function of *PtrBTB82* in *P. trichocarpa*, we constructed a plant expression vector containing *PtrBTB82* and overexpressed it using the cauliflower mosaic virus 35S (CaMV35S) promoter. After genomic DNA PCR, RT-PCR verification and western blotting analysis (Figure 4A and Figure S3), three overexpression lines *PtrBTB82OE*-1/4/5 were chosen for functional studies because they possessed more than 200-fold higher *PtrBTB82* transcription levels than non-transgenic WT plants.

Next, we observed the phenotypes of WT plants and 3-month-old overexpression lines in the greenhouse. The *PtrBTB82OE*-1/4/5 lines showed significant morphological alterations in comparison to WT (Figure 4B). Plant height, the number and length of stem internodes did not significantly differ between *PtrBTB82OE*-1/4/5 and WT plants (Figure 4B–E). In contrast to WT, the three overexpression lines’ basal stem diameters decreased by 16%, 13%, and 11%, respectively (Figure 4F), suggesting that *PtrBTB82* inhibits stem diameter growth in *P. trichocarpa*.

### 2.4. Overexpression of PtrBTB82 Decreases Xylem Width and Cambium Activity

**Figure 5:**
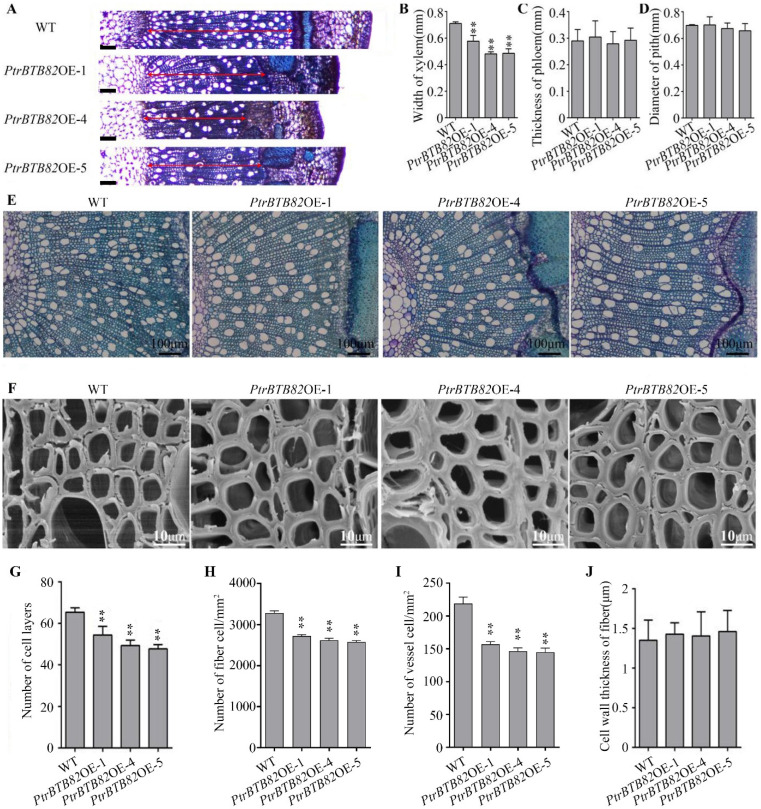
Analysis of stem morphology and structure in *PtrBTB82*-overexpressing *P. trichocarpa*. (**A**) Light microscopic observations of cross-sections from the 12th stem internodes of 3-month-old wild-type (WT) and *PtrBTB82OE-*1/4/5 overexpression lines. Scale bar: 100 μm. (**B**–**D**) Statistical analysis of width of xylem, thickness of phloem, diameter of pith. Values are means ± SDs (n = 3). Asterisks denote a significant difference from WT using the Student′s *t*-test: ** *p* < 0.01. (**E**) Magnification of the cross-sections from (**A**). Red arrows indicate the width of secondary xylem. (**F**) SEM of the 12th stem internodes from WT and *PtrBTB82* overexpressing lines. (**G**) Statistics of the number of xylem cell layers using light microscopic images from the (**E**). (**H**,**I**) Statistics of the number of fiber and vessel cells per cross-sectional area (mm^2^). (**J**) Wall thickness of mature xylem fibers using the SEM images from the (**F**). Values are means ± SDs (n = 3 (**G**–**I**), and 100 (**J**)). Asterisks denote a significant difference from WT using the Student′s *t*-test; ** *p* < 0.01.

We further explored the role of *PtrBTB82* in *P. trichocarpa* stem growth. The 12th stem internodes from 3-month-old WT and overexpression plants were cross-sectioned, and the sections stained with toluidine blue were examined under a light microscope. The results revealed that *PtrBTB82* overexpression lines were drastically reduced from pith to periderm of stem cross-sections when compared to WT plants (Figure 5A). Furthermore, xylem width in *PtrBTB82OE*-1, -4, and -5 was reduced by 17%, 30%, and 29%, respectively, compared to WT, whereas no significant differences were found in the pith and phloem tissues (Figure 5B–D). Next, we conducted a statistical analysis of the number of secondary xylem cell layers and found that *PtrBTB82OE*-1, -4, and -5 had 13%, 20%, and 21% less layers than WT (Figure 5G). Statistical analysis revealed that the number of xylem fiber and vessel cells per unit area in *PtrBTB82* overexpression lines was much lower than in WT plants (Figure 5H,I), indicating a considerable increase in secondary xylem cell size. Scanning electron microscopy (SEM) analysis revealed no significant changes in SCW thickness of mature xylem fibers between WT and overexpression plants (Figure 5J).

**Figure 6:**
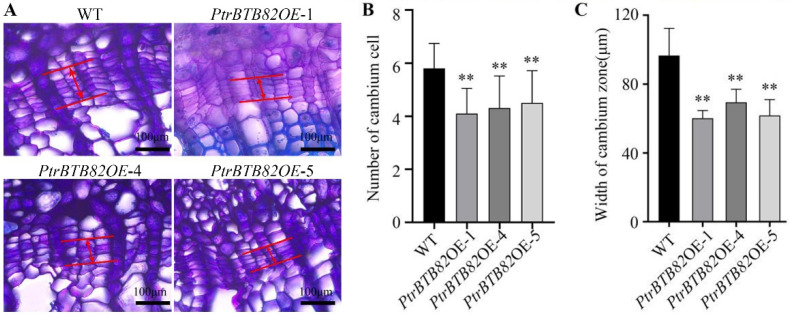
Overexpression of *PtrBTB82* reduces vascular cambial activity. (**A**) Light microscopic images of the 6th stem internode cross-sections from 3-month-old wild-type (WT) and *PtrBTB82*-overexpressing lines. Red solid lines with arrows connecting two red solid lines represented cambial zones. (**B**) Statistical analysis of the number of cambial cell layers in each file of WT and overexpressing lines using the light microscope from (**A**). The values are means ± SDs (n = 110). (**C**) Statistics of cambial zone width obtained with the light microscope from (**A**). The values are means ± SDs (n = 3). Asterisks indicate a significant difference from WT using the Student’s *t*-test (** *p* < 0.01).

To better understand the decrease in xylem width of *PtrBTB82* overexpression plants, we investigated their vascular cambium. The vascular cambium in wild-type *P. trichocarpa* typically exhibited six cell layers, whereas overexpression lines had four or five (Figure 6A,B). The number of cambial cell layers differed between WT and *PtrBTB82* overexpression plants, indicating that *PtrBTB82* reduces cambial activity. Statistical analysis revealed that the width of the cambium zone in overexpression lines was reduced by 38%, 28%, and 36%, respectively, when compared to the WT (Figure 6C). Taken together, our data indicate that *PtrBTB82* suppresses secondary xylem growth by repressing cambial activity.

### 2.5. Overexpression of PtrBTB82 Alters Wood Chemical Composition in Poplar

**Figure 7:**
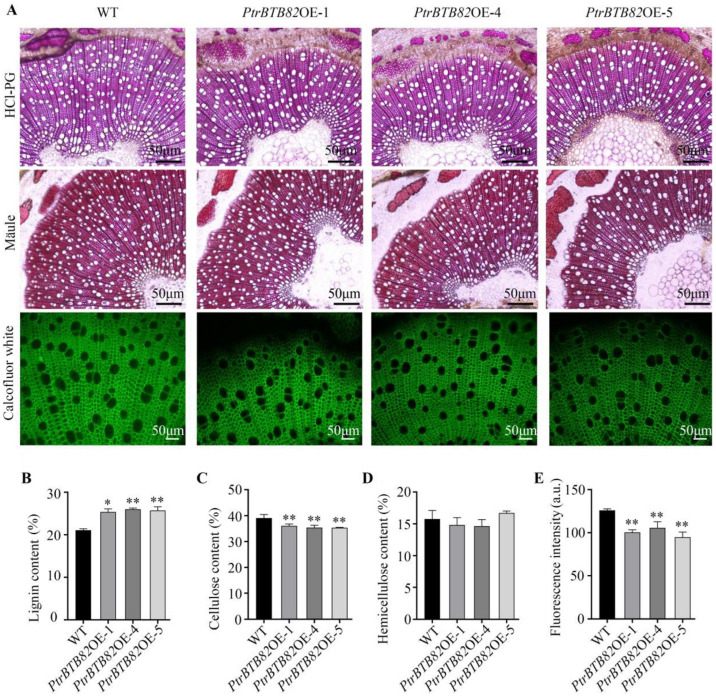
Analysis of lignin, cellulose, and semi-cellulose content in *PtrBTB82* overexpression wood. (**A**) A microscopic examination of cross-sections from the 12th stem internode of wild-type (WT) and overexpression lines. The cross-sections were stained with phloroglucinol-HCl, Mäule solution, and calcofluor white to determine lignin, S-lignin, and cellulose content. The green color represented fluorescent signals extracted from calcofluor white-stained cellulose using ZEN 3.1 software. (**B**–**D**) Statistics for lignin, cellulose, and hemicellulose content in *PtrBTB82* overexpression wood. The values are means ± SDs of three replicates (n = 3). (**E**) Fluorescence intensity data extracted from calcofluor white-stained cellulose. The values are the means ± SDs of three replicates (n = 50). Asterisks indicate a significant difference from WT using the Student’s *t*-test: * *p* < 0.05, ** *p* < 0.01.

We investigate the effects of *PtrBTB82* overexpression on wood composition using phloroglucinol-HCl, Mäule and calcofluor white histochemical staining, which estimates total lignin, mono-lignin, and cellulose content, respectively. *PtrBTB82* overexpression lines exhibited slightly darker staining with phloroglucinol-HCl than WT does, while mono-lignin staining intensity was nearly identical in both (Figure 7A). Conversely, calcofluor white staining revealed a slight decrease in fluorescence intensity in the overexpression lines compared to WT (Figure 7A). Analysis of quantifying fluorescence intensity using ImageJ software (v1.58j) showed that the average cellulose fluorescence intensity in the overexpression lines decreased by 20.3%, 16.1%, and 24.6%, respectively, compared to WT (Figure 7E). These findings suggest that overexpression of *PtrBTB82* increases lignin content and reduces cellulose content in wood.

Next, the concentrations of lignin, cellulose, and hemicellulose were determined in the wood of WT and overexpression lines. In comparison to the WT, total lignin content in *PtrBTB82OE*-1, -4, and -5 increased by 20%, 23%, and 22%, respectively, whereas cellulose content reduced by 7.7%, 9.5%, and 9.6% (Figure 7B,C). However, hemicellulose content did not differ significantly between WT and overexpression wood (Figure 7D). Overall, histochemical staining and quantitative measures show that overexpression of *PtrBTB82* can affect lignin and cellulose levels in wood.

### 2.6. Expression of the Genes Associated with Cambium Activity in PtrBTB82-Overexpressing Poplars

**Figure 8:**
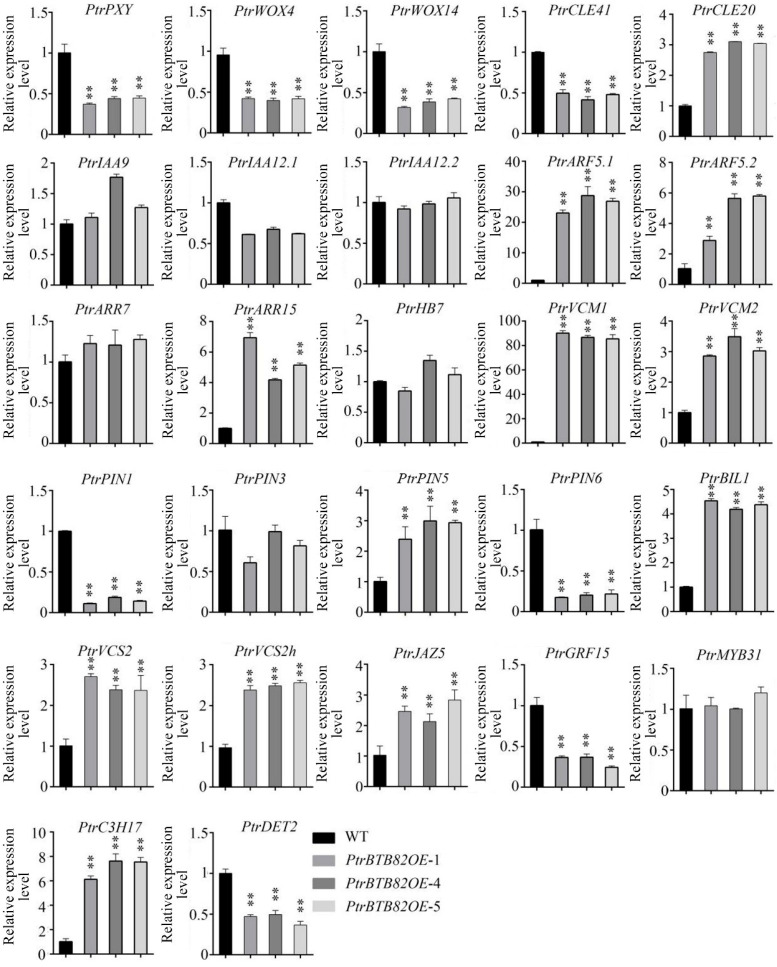
Expression of the genes associated with cambial activity in *PtrBTB82*-overexpressing *P. trichocarpa*. Cambial tissues of wild-type (WT) and *PtrBTB82* overexpression lines were used to isolate total RNAs for RT-qPCR analysis. These genes include WUSCHEL-related pathway *(PtrCLE41*, *PtrPXY*, *PtrWOX4*, and *PtrWOX14*), plant hormone signaling (*PtrIAA9/12*, *PtrARF5*, *PtrPIN1/3/5/6*, *PtrARR7/15*, *PtrBIL1*, *PtrDET2*, and *PtrJAZ5*), as well as transcriptional regulators and peptide (*PtrHB7*, *PtrVCM1/2*, *PtrVCS2/2h*, *PtrGRF15*, *PtrMYB31*, and *PtrC3H17*). A change of more than twofold with the value *p* < 0.05 was used to determine differential expression. Values are means ± SDs (n = 3). Asterisks denote a significant difference from WT using the Student’s *t*-test: ** *p* < 0.01.

To investigate the reason for reduced cambium activity in the overexpression lines, we examined whether the expression of some genes associated with cambial activity had changed using RT-qPCR analysis. The 27 genes (Figure 8) were selected on the basis of functional annotations, mainly in hormone signaling and multilayered transcriptional networks. The *CLE41-PXY-WOX4/14* signaling module, a key regulator of cambial cell division and maintenance, was markedly downregulated at transcriptional levels, indicating inhibited cambial cell proliferation in *PtrBTB82*-overexpressing poplars. *CLE20*, potentially repressing *WOX4* expression via an intermediate receptor [46], was significantly upregulated, potentially enhancing the inhibition of cambial activity in the overexpression lines. The auxin transport genes *PIN1* and *PIN6* were synchronously downregulated, further suggesting suppression of cambial cell proliferation. Meanwhile, transcript levels of *ARF5.1*, *ARF5.2*, and *ARR15* were significantly increased; the upregulation of the *ARF5-ARR15* module likely inhibits cytokinin biosynthesis, possibly repressing *PIN1* transcription. Additionally, *PIN5* and its upstream regulators *VCM1* and *VCM2* were significantly upregulated, which contribute to the inhibition of cambial activity [47]. Some transcription factors *VCS2/2h* and *C3H17* known to suppress cambial activity and *BIL1* exhibited significantly increased expression in *PtrBTB82*-overexpressing poplar [48,49]. As an inhibitor of cambium activity [50], the jasmonic acid (JA)-related gene *JAZ5* was significantly upregulated. The brassinosteroid (BR) biosynthesis gene *DET2* was significantly downregulated, suggesting a reduction in BR-mediated cell expansion [51]. In contrast, the *IAA9/12-ARR7* regulatory module and its downstream targets, including *HB7*, *PIN3*, and *MYB31*, remained stably expressed. These data indicate that *PtrBTB82* should involve in cambial activity of *P. trichocarpa*.

### 2.7. Expression of Lignin, Cellulose and Semi-Cellulose Synthesis Genes in PtrBTB82-Overexpressing Poplars

To determine how *PtrBTB82* affects wood SCW synthesis, transcription levels of lignin, cellulose, and hemicellulose synthesis genes were analyzed in *PtrBTB82* overexpressing lines. In poplar, whole series of lignin biosynthesis enzymes were encoded by the 22 genes, i.e., *PtrPAL1/2/3/4/5*, *PtrC4H1/2*, *Ptr4CL3/5*, *PtrHCT1/6*, *PtrC3H3*, *PtrCCoAOMT1/2/3*, *PtrCCR2*, *PtrF5H1/2*, *PtrCOMT2*, *PtrCAD1*, and *PtrCSE1/2*, with high expression levels in secondary xylem [52].

**Figure 9:**
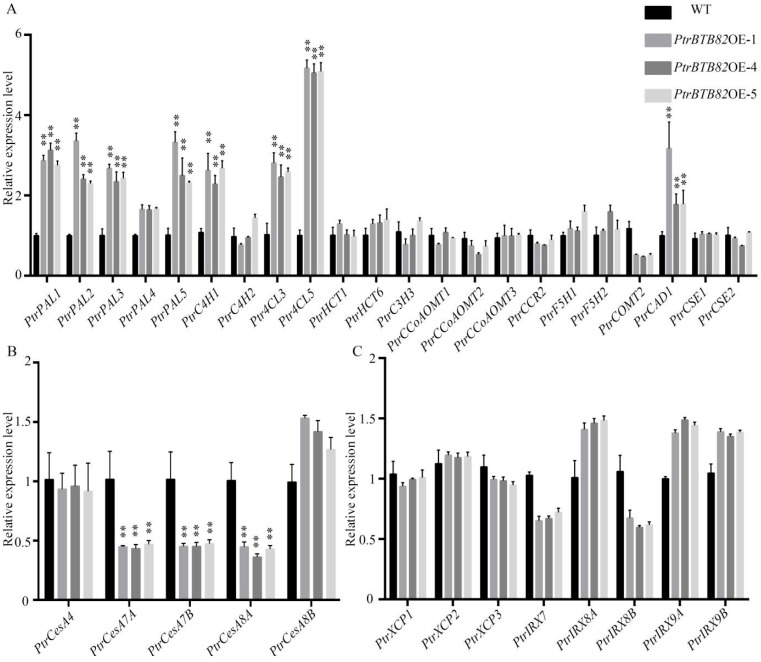
Expression of lignin, cellulose and hemicellulose biosynthesis genes in *PtrBTB82*-overexpressing *P. trichocarpa*. (**A**–**C**) Lignin, cellulose and hemicellulose biosynthesis genes, respectively. Developing xylem of wild-type (WT) and *PtrBTB82* overexpression lines was used to isolate total RNAs for RT-qPCR analysis. A change of more than 2-fold with the value *p* < 0.05 was used to determine differential expression. Values are means ± SDs (n = 3). Asterisks denote a significant difference from WT using the Student’s *t*-test: ** *p* < 0.01.

Our RT-qPCR analysis revealed that *PtrPAL1/2/3/5*, *PtrC4H2*, and *Ptr4CL3/5* were upregulated more than 2-fold compared to WT in the overexpression lines at transcription levels (Figure 9A). Except for *PtrCAD1*, the majority of lignin biosynthetic downstream enzyme genes did not differ significantly between WT and overexpression plants (Figure 9A). For SCW cellulose synthesis genes, *PtrCesA7A/B* and *PtrCesA8A* transcript levels were dramatically reduced in the overexpression lines, whereas *PtrCesA4* and *PtrCesA8B* transcript levels remained significantly unchanged (Figure 9B). In contrast, we found no significant changes in the expression of key hemicellulose biosynthesis genes such as *PtrXCP1/2/3*, *IRX7*, *IRX8A/B*, and *IRX9A/B* (Figure 9C). Our findings are consistent with the notion that overexpression of *PtrBTB82* increases lignin content and reduces cellulose content in wood of transgenic poplars.

## 3. Discussion

In this study, we identified 103 *BTB* family genes in *P. trichocarpa*, slightly more than the 95 genes reported previously [44]. This discrepancy is likely due to continuous updates and refinements in protein databases, reflecting the dynamic nature of genome annotation. Generally, gene expression patterns are closely associated with biological functions, offering valuable clues about their potential roles and regulatory mechanisms. Expression profiling using the eFP browser revealed highly diverse transcriptional patterns of *BTB* members across roots, internodes, and leaves, suggesting functional diversification within this gene family. Among these genes, we identified an only-BTB subgroup member, *PtrBTB82*, that exhibited a high expression in xylem. GUS staining further confirmed its predominant activity in the secondary vascular tissues (Figure 2 and Figure 3). These results suggest that *PtrBTB82* plays the role in cambial activity and SCW formation during stem development. Consistently, overexpression of *PtrBTB82* in *P. trichocarpa* led to reduced cambial activity and consequently a narrower xylem compared with the WT (Figure 5 and Figure 6). In addition to *PtrBTB82*, we observed high expression of *PtrBTB79* in internodes and roots. But they are distributed across different subfamilies and shares limited protein similarities, suggesting a lower possibility in analogous functions. Whether it exerts overlapping or distinct functions with *PtrBTB82* in poplar remains to be determined in the future.

Cambial activity directly determines the extent and rate of wood formation. Previous studies have demonstrated that changes in phytohormone concentrations directly affect cambial activity, with the *IAA9/12-ARF5-HB7* pathway being a major regulator of auxin distribution [5]. In our study, the transcriptional activity of *IAA9/12* and *HB7* did not change significantly, but the expression of *ARF5* was altered (Figure 8). One possible explanation is the involvement of *BIL1* (*BIN2-LIKE1*), which acts as a member of the GSK3-like kinase and a critical regulator in the brassinosteroid signaling pathway [53,54]. In addition to this role, *BIL1* has been shown to enhance the inhibitory effect of *ARF5* on cambial activity, and to influence downstream cytokinin signaling by activating a set of negative regulators, including *ARR7/15* [55]. In our study, we found that the transcript levels of *BIL1*, *ARF5*, and *ARR15* were all significantly increased in the *PtrBTB82* overexpression lines. Speculatively, *PtrBTB82* overexpression reduced cambial activity, possibly through the complex interplay of auxin, brassinosteroid, and cytokinin signaling pathways, rather than solely by altering auxin biosynthesis.

In addition to hormonal signaling, transcriptional regulation is essential for maintaining cambial activity. Previous studies have demonstrated that the *NAC* transcription factor *WOX4* serves as a central regulator, not only by integrating multiple hormone signaling pathways but also by directly modulating cambial activity through the WUSCHEL-related pathway [56]. In this study, we found that *CLE41*, *PXY*, *WOX4*, and its homolog *WOX14* were significantly downregulated in the *PtrBTB82* overexpression lines. This observation suggests that *PtrBTB82* should involve in the WUS-mediated pathway to influence cambial activity. *CLE41* peptide is synthesized in the phloem and functions as the upstream signal of this pathway [57]. Although low-level expression of *PtrBTB82* in the phloem (Figure 2 and Figure 3C,D), such low but spatially precise expression is presumptively operative to modulate *CLE41* transcription. The upregulation of *CLE20*, a CLE peptide known to indirectly repress *WOX4* expression, further supports this hypothesis [46]. Moreover, the *CLE41/44-PXY* module has been shown to suppress *BIL1* and integrate brassinosteroid signaling into the auxin pathway to regulate cambial activity [55]. This explains why *BIL1* is upregulated in the overexpression lines, and it’s further proven that *PtrBTB82* overexpression reduces cambial activity by modulating *CLE41* expression and affecting downstream signaling pathways. It is proposed that *PtrBTB82* might act on the *CLE41–PXY-WUS* signaling complex, which modulates the complex interplay of auxin, brassinosteroid, and cytokinin signaling pathways.

Plant cell walls are generally classified into primary and secondary walls. In fibers and vessel elements of the trees, the SCW is a key structure that provides mechanical support. It is mainly composed of cellulose, hemicellulose and lignin, whose biosynthesis requires the coordinated expression of related genes [19]. Typically, changes in cellulose and/or lignin content may lead to the alteration in SCW thickness. *PtrBTB82* overexpression significantly increased lignin content and decreased cellulose content (Figure 7), but the thickness of the SCWs remained unchanged. Previous study has shown that *C4H1*/*2* inhibited transgenic poplar trees with reduced lignin content does not significantly change the thickness of the fiber wall [58], which is consistent with our observations. Changes in chemical composition of SCWs are usually attributable to transcriptional regulation in their biosynthetic genes. In *PtrBTB82* overexpression lines, lignin biosynthetic genes, including *PAL, C4H*, *4CL*, and *CAD1*, were upregulated, leading to elevated lignin accumulation. The expression of key S-lignin biosynthetic genes (*PtrF5H1/2* and *PtrCOMT2*) remains significantly unchanged and Mäule staining for detecting S-lignin monomer reveals no obvious differences between WT and overexpression lines (Figure 7A and Figure 9). In light of these findings, G- or H-lignin might increase in overexpression lines, possibly producing the potential shift in S/G ratio.

Meanwhile, the expression of SCW *CESA7A/B* and *CESA8A* was significantly downregulated in *PtrBTB82* overexpression lines, explaining the reduced cellulose content. SCW *CesA* genes are known to be highly conserved across vascular plants [59,60,61], and mutations in any SCW *CesA* gene typically lead to severe reductions in cellulose content and SCW thickness, accompanied by weakened stem mechanical strength and irregular xylem phenotypes, such as vessel collapse [61,62]. However, our results revealed a decline in cellulose content without a significant reduction in wall thickness (Figure 5J). It may be due to a compensatory effect among wall components, in which excess lignin deposition offsets the adverse impact of reduced cellulose on wall thickness. Since lignin and hemicellulose are closely associated and crosslinked during SCW formation [63], enhancing their interactions might help maintain cell wall integrity even under conditions of reduced cellulose content. In addition, the increased transcript levels of lignin biosynthesis genes together with the pronounced repression of SCW *CESA* genes, both of which are well-established downstream targets of the *NAC-MYB* master regulatory cascade [21], raise the question of whether these transcriptional changes are mediated directly by *PtrBTB82* remains to be elucidated in the future.

BTB/POZ proteins constitute one of the largest families of substrate adaptors for CULLIN3-based E3 ubiquitin ligase complexes in plants [40]. The RT–qPCR results clearly reveal the broad impact of *PtrBTB82* overexpression on downstream gene networks on cambial activity (Figure 8). PtrBTB82, as a member of the only-BTB subfamily, contains only a conserved BTB/POZ domain and have no obvious DNA-binding domain. It raises a question of whether it can directly interact with CUL3 and subsequently target yet-unknown positive regulators of cambial activity or SCW formation for degradation. If such, its regulatory mechanism cannot be elucidated solely through RT–qPCR analysis. Therefore, future studies employing yeast two-hybrid assays and related approaches will be required to verify whether *PtrBTB82* functions in this manner.

In summary, *PtrBTB82* is an only-BTB type BTB/POZ gene predominantly expressed in secondary xylem. Overexpression of *PtrBTB82* in *P. trichocarpa* suppressed the expression of genes involved in the WUSCHEL-related pathway and plant hormone signaling, thereby reducing cambial activity and inhibiting xylem development. In addition, overexpression of *PtrBTB82* led to upregulated genes associated with lignin biosynthesis (*PtrPALs*, *PtrC4H1*, *Ptr4CL* and *PtrCAD1*) while downregulating SCW *CesA* genes (*PtrCESA7A/B* and *PtrCESA8A*), which resulted in significant alterations in the chemical composition of wood.

## 4. Materials and Methods

### 4.1. Plant Materials and Growth Conditions

*P. trichocarpa* genotype *Nisqually-1* was used as the plant material in this study. Sterile plantlets were propagated using in vitro tissue culture methods [64]. The WT and transgenic plant lets were grown in soil for phenotypic analysis in a greenhouse (25 to 28 °C; 16/8 h light/dark cycle) with a light intensity of ~250 μmol·m^−2^·s^−1^.

### 4.2. Identification of the BTB Family Genes in P. trichocarpa

We employed two complementary approaches to identify potential BTB proteins in *P. trichocarpa*. (1) The potential BTB proteins were identified in *P. trichocarpa* using the Hidden Markov Model (HMM) profiles for the conserved BTB/POZ domains (PF00651) from the InterPro database (https://www.ebi.ac.uk/interpro/; accessed on 8 March 2024). The protein sequences, genomic sequences, and coding sequences (CDS) of all BTB genes were obtained from the *P. trichocarpa* v4.1 genome database [65]. (2) We searched the InterPro database for BTB/POZ domains (IPR000210, IPR011333, and IPR016073) and retrieved the corresponding conserved motifs. These motifs were subsequently used to perform BLAST searches against the *P. trichocarpa* genome in the Phytozome database (https://phytozome-next.jgi.doe.gov/blast-search; accessed on 9 March 2024). The resulting candidate genes from two independent BLAST searches were compared, and redundant sequences were removed to obtain a non-redundant BTB gene set.

### 4.3. Construction of the BTB Family Phylogenetic Tree, Gene Structure and Motif Analysis

The phylogenetic tree of 103 PtrBTB proteins was constructed using the neighbor-joining (NJ) method with 1000 bootstrap replicates and p-distance in MEGA11 after alignment and trimming of amino acid sequences. The tree was then beautified using the iTOL online platform (https://itol.embl.de/; accessed on 25 May 2024). Gene structures, including the organization of exon and intron, and conserved motifs, were generated with TBtools-II software (v2.310) [66]. Conserved motifs of the *PtrBTB* genes were analyzed by the Multiple Expectation Maximization for Motif Elucidation (MEME) system (https://meme-suite.org/meme/tools/meme; accessed on 10 March 2024) [67].

### 4.4. Analysis of Electronic Expression Data

Tissue-specific expression data on the *PtrBTBs* were downloaded from the *Populus* eFP browser (http://bar.utoronto.ca/efppop/cgi-bin/efpWeb.cgi; accessed on 14 March 2024). The heatmap was generated using TBtools-II software (v2.310). Under the “Graphics” tab, the “Heatmap Illustrator” module was selected, and the downloaded expression data along with the corresponding gene IDs were imported. The dataset was normalized across all samples, and the color scale was adjusted to a range from 0 to 1 [66]. Stem tissue expression data and line charts were directly generated from the AspWood electronic expression database (http://aspwood.popgenie.org; accessed on 26 May 2024), and the underlying data were obtained from the study published by Sundell et al. [68].

### 4.5. RNA Extraction and RT-qPCR

For determination of *PtrBTB82* transcriptional levels in various tissues of *P. trichocarpa*, apical buds (AB), young leaves (YL), mature leaves (ML), phloem (Ph), developing xylem (Xy), and roots (Rt) were collected from 4-month-old WT. Three biological replicates were performed for each tissue, with each replicate consisting of a pooled sample from three WT trees. For the identification of transgenic lines, developing xylem tissues were collected from WT and transformants grown in the greenhouse. For the analysis of expression of the genes associated with cambial activity and SCW biosynthesis, the rich cambial tissues and developing xylem were collected from 4-month-old WT and transgenic lines. Three biological replicates were performed, each consisting of pooled samples from three individual trees. All samples were immediately frozen in liquid nitrogen and ground into a fine powder.

Total RNA was extracted using the pBIOZOL reagent (BioFlux, Hangzhou, China). First-strand cDNA was synthesized using the PrimeScript RT reagent kit with gDNA Eraser (TaKaRa, Beijing, China), following the manufacturer’s instructions. RT-qPCR was conducted using an ABI 7500 Real-Time PCR System (Applied Biosystems, Waltham, MA, USA) and TB Green Premix Ex Taq II (TaKaRa, Beijing, China), each 20 µL reaction mixture contained 10 µL of 2× TB Green Premix Ex Taq II (Tli RNaseH Plus), 1 µL of cDNA template, 0.4 µL of ROX Reference Dye II, 0.8 µL of each gene-specific primer, and 7 µL of distilled water. Relative transcript abundance was calculated using the 2^−ΔΔCT^ method and normalized to the expression of *PtrActin2*. Each assay was performed with three technical replicates. The gene-specific primers used for RT-qPCR are listed in Supplementary Table S1.

### 4.6. Vector Constructs

Genomic DNA was extracted from leaves of 3-month-old wild-type plants using a Plant Genomic DNA Extraction Kit (Bioteke, Beijing, China). For the pCAMBIA1300-*ProPtrBTB82*::GUS construct, the 1815 bp promoter regions of *PtrBTB82* genes were amplified with specific primers, and the purified DNA fragments after double digestions with *Sac*I and *Xba*I endonucleases were ligated into pCAMBIA1300 vector containing a GUS gene. For the pCAMBIA1300-35s::*PtrBTB82*-3xflag construct, the CDS of *PtrBTB82* gene was ligated into the pCAMBIA1300 (double digestions with *BamH*I and *Sal*I endonucleases) vector under the control of the CaMV 35S promoter. All constructs were verified by DNA sequencing. The primers used for vector construction are listed in Supplementary Table S1.

### 4.7. Generation of Transgenic P. trichocarpa

The constructed pCAMBIA1300-*ProPtrBTB82*::GUS and pCAMBIA1300-35S::*PtrBTB82*-3xflag vectors were introduced into the *Agrobacterium tumefaciens* strain GV3101 for genetic transformation of *P. trichocarpa*. The *Agrobacterium*-mediated transformation method was performed as described by Li and Zhen et al. [64]: Healthy 1-month-old wild-type *P. trichocarpa* tissue culture seedlings were used as the transformation material, with hygromycin (Hyg) as the selectable marker. The *Agrobacterium* carrying the corresponding vectors was incubated to an OD600 = 0.6. The bacterial pellet was collected by centrifugation at 2200× *g* for 10 min at 4 °C and resuspended in 50 mL of suspension solution (containing 0.04 g WPM, 1.25 g sucrose, 0.0136 g MES, pH = 5.2). The stems of *P. trichocarpa* seedlings were cut into segments approximately 1 cm in length and immersed in the suspension solution for infection with gentle shaking for 20 min. The infected explants were co-cultivated for 48 h. The stem explants were then transferred to a selection medium containing 10 mg L^−1^ hygromycin under light conditions. After 25 d, the stem explants were transferred to a selection medium containing 5 mg L^−1^ hygromycin for further cultivation. After 20 d, resistant buds were induced. The hygromycin-resistant shoots were excised and placed on a rooting medium containing 5 mg L^−1^ hygromycin.

### 4.8. GUS Staining

GUS staining analysis was performed on 1-month-old *proBTB82*::GUS transgenic seedlings grown in tissue culture bottles and 3-month-old trees grown in soil, with six independent lines analyzed to ensure the reliability of the results. Wild-type plants were used as negative controls, and the experiments were repeated three times. Various tissues were incubated in the GUS staining solution [0.1 M Na_3_PO_4_ buffer (pH 7.0), 10 mM EDTA, 2 mM K_3_[Fe(CN)_6_], 2 mM K_4_[Fe(CN)_6_], 1 mM X-Gluc, and 0.1% (*v*/*v*) Triton X-100] at 37 °C for 4 h. After GUS signal development, chlorophyll was removed from the samples multiple times using 75% (*v*/*v*) ethanol. Images of stem sections were captured using a EX21 upright microscope (Soptop, Ningbo, China); other tissue images were recorded using an SZX7 stereomicroscope (Olympus, Tokyo, Japan).

### 4.9. Protein Extraction and Western Blot Analysis

Developing xylem samples were collected from the stems of 4-month-old WT and *PtrBTB82* overexpression lines. Three biological replicates were performed, each consisting of pooled samples from three individual trees. Plant materials were rapidly frozen in liquid nitrogen and ground into a fine powder. The samples were homogenized on ice for 1 h in protein extraction buffer (100 mM Tris–HCl, pH 8.0, and 1% SDS), followed by boiling for 10 min. After centrifugation at 14,000 rpm for 10 min, the supernatants (protein extracts) were collected for sodium dodecyl sulfate-polyacrylamide gel electrophoresis (SDS-PAGE) analysis. Total proteins were separated on 10% SDS-PAGE gels and transferred onto polyvinylidene fluoride (PVDF) membranes. The membranes were blocked at 4 °C overnight and then incubated for 1 h with the anti-FLAG primary antibody (1:10,000; M20008, Abmart, Shanghai, China) and the horseradish peroxidase (HRP)-conjugated secondary antibody Anti-Mouse IgG H&L (1:10,000; AB6789, Abcam, Cambridge, UK). Signals were detected using the Enhanced Chemiluminescence (ECL) Western Blotting Substrate (34579, Thermo Fisher Scientific, Waltham, MA, USA) and captured on X-ray film.

### 4.10. Histological Staining and Microscopy Analyses

For light microscopy, 3-month-old *PtrBTB82*-overexpressing (OE) plants and corresponding wild-type (WT) plants were subjected to histological analysis. Stem segments from the 6th and 12th internodes of WT and OE plants were sectioned using a vibratome (Leica VT1200S; Leica, Wetzlar, Germany) at a thickness of 70 μm. The sections were stained with 0.05% or 0.01% (*w*/*v*) toluidine blue O (TBO) solution, or with 1% phloroglucinol-HCl solution. Images of the stem sections were captured using a EX21 upright microscope (Suptop, China).

For quantification of the number of cambial cell layers in WT and *PtrBTB82*-overexpressing lines, stem segments at the 6th internode were collected from 3-month-old WT and *PtrBTB82* overexpression lines. Stem sections were prepared using the method described above and stained with 0.01% (*w*/*v*) toluidine blue O (TBO) solution for microscopic observation. Well-preserved and clearly defined cambial regions were selected for analysis. Three trees from each line were analyzed, and the experiments were repeated three times. Each replicate included 110 measurements of the number of cambium cell layers.

For fluorescence microscopy, stem segments from the 12th internode of WT and OE plants were sectioned using a vibratome (Leica VT1200S; Leica) at a thickness of 70 μm. Lignin autofluorescence in cross-sections was imaged under the DAPI channel using an Axio Imager.A2 microscope (Zeiss, Oberkochen, Germany). Cellulose was stained with 0.01% (*v*/*v*) calcofluor white solution and imaged under the DAPI channel with the same microscope.

For scanning electron microscopy (SEM), free-hand cross-sections of stem segments from the 12th internode of 3-month-old OE and WT plants, grown under normal or inclined conditions, were coated with gold (Au) at 10 mA for 60 s, transferred to an SEM chamber (JCM-5000, Nanotech, Tokyo, Japan), and imaged to analyze secondary wall thickness. Three trees from each line were analyzed, and the experiments were repeated three times. Each replicate included 100 measurements of mature xylem fiber wall thickness (approximately 20–25 fiber layers adjacent to the cambium).

### 4.11. Wood Composition Assay

For the determination of lignin and crystalline cellulose content, samples were prepared as follows: the basal stems of 4-month-old WT and transgenic trees grown in the greenhouse were peeled, air-dried at 55 °C, and ball-milled into a fine powder. The powder was then sequentially washed with 70% aqueous ethanol, a chloroform/methanol (1:1, *v*/*v*) solution, and acetone. The resulting insoluble residues were used as cell wall materials for the assays of crystalline cellulose and lignin content. The crystalline cellulose and lignin contents were measured according to the methods described by Foster et al. [69,70]. Hemicellulose content was assayed according to the instructions provided by the kit manufacturer (Geruisi Biotechnology, Suzhou, China; http://geruisi-bio.com).

### 4.12. Statistical Analysis

All data analyses and statistical tests were conducted by GraphPad Prism version 10.4. Student’s *t*-test and ANOVA were used to determine the statistical significance between the mutant and WT samples. Values are displayed as the means ± standard deviation (SD), and the number of asterisks indicates statistical significance at different levels (* *p* < 0.05, ** *p* < 0.01).

## Data Availability

All experimental data are provided in the main text and Supplementary Materials.

## Supplementary Materials

The following supporting information can be downloaded at: https://www.mdpi.com/article/10.3390/plants15010068/s1.
