# Supplementary material for: Overexpression of a Xylem-Dominant Expressing BTB Gene, PtrBTB82, Influences Cambial Activity and SCW Synthesis in Populus trichocarpa
Source: Plants (Basel). 2025 Dec 25;15(1):68. doi: 10.3390/plants15010068 (PMC12787359; doi:10.3390/plants15010068)
Supplement: Supplementary file 1 [file plants-15-00068-s001.zip › Figure S1-S3.pdf]

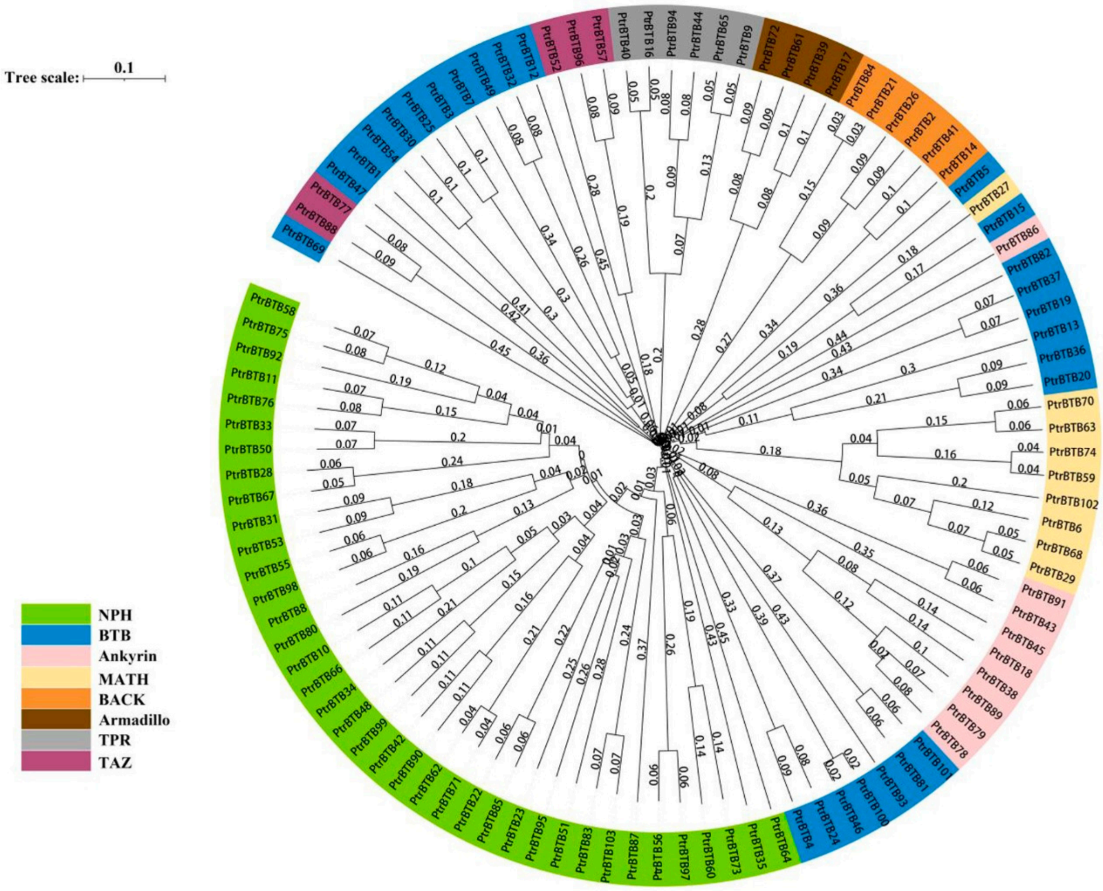

Figure S1. Phylogenetic tree of BTB proteins from *P.trichocarpa*. Analysis classified the 103 BTB proteins identified in *P.trichocarpa* into eight subgroups: NPH, BTB-only, Ankyrin, MATH, BACK, Armadillo, TPR, and TAZ. A phylogenetic tree was constructed using the neighbor-joining (NJ) method implemented in MEGA11, with 1000 bootstrap replicates to assess branch reliability.

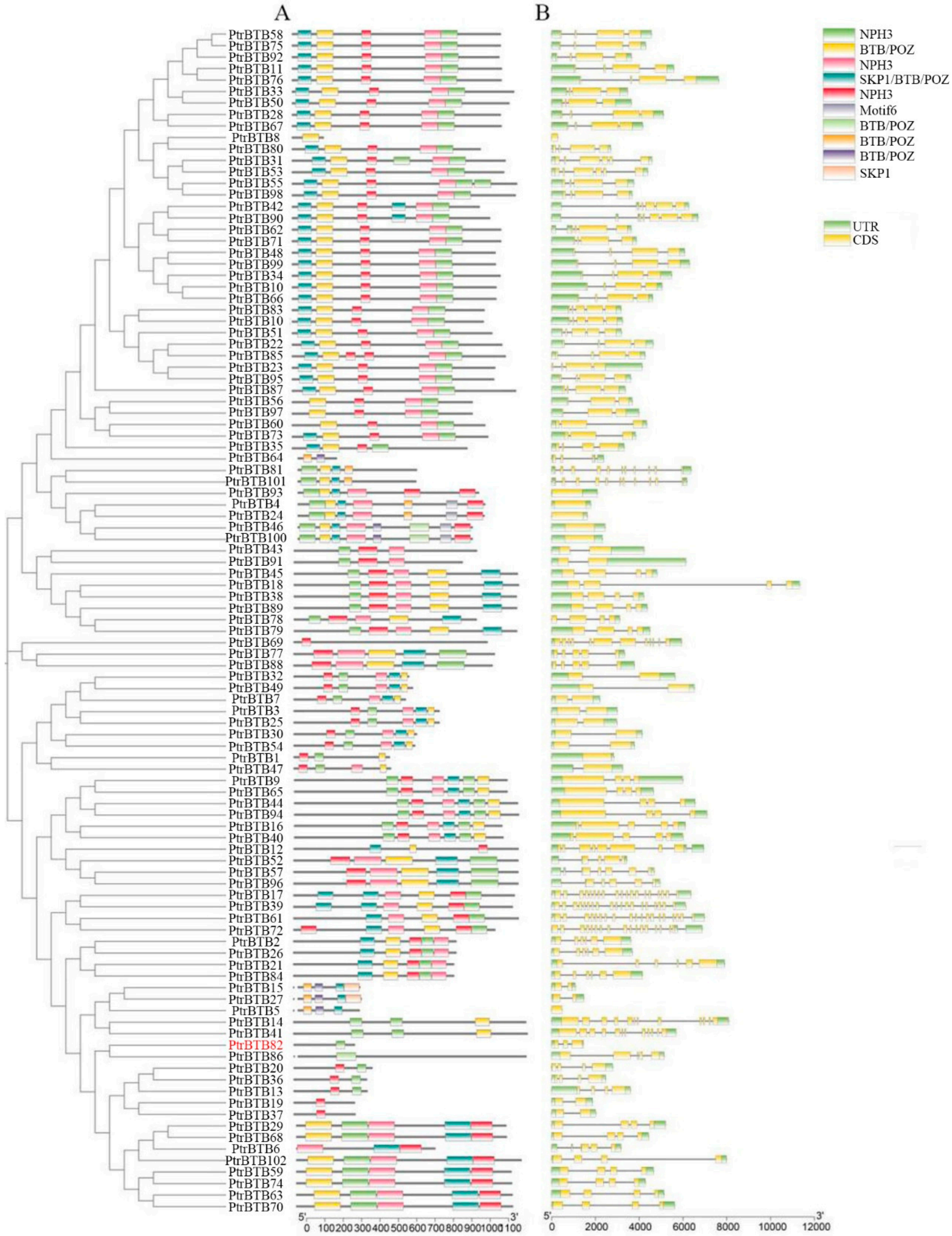

Figure S2. Gene structure and protein structure of *PtrBTB* gene family. (A) Protein motifs. Colored boxes indicate conserved motifs, and black lines indicate non-conserved regions. (B) Gene structure. Yellow boxes indicate exons, black lines indicate introns, and green boxes indicate untranslated regions (UTRs). The scale bar at the bottom provides a reference for exon and intron length. The gene highlighted in red indicate that selected for this study.

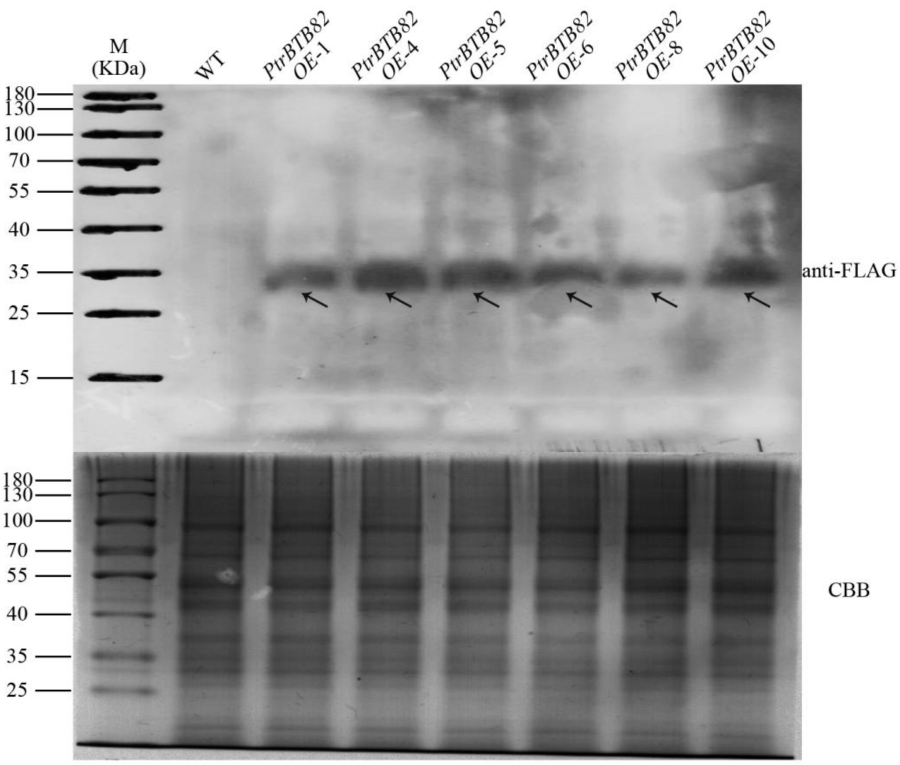

Figure S3. Total proteins were extracted from the xylem of WT, *PtrBTB82OE*-1, -4, -5, -6, -8, and -10 transgenic lines. After separation on a 10% SDS–PAGE gel, immunoblotting was performed using an anti-FLAG antibody. A representative Coomassie Brilliant Blue (CBB)–stained gel is shown to confirm equal loading. The black arrow indicates the approximate position of the PtrBTB82–3×FLAG fusion protein, which has a molecular weight of ~34 kDa.
