# Supplementary material for: Overexpression of a Xylem-Dominant Expressing BTB Gene, PtrBTB82, Influences Cambial Activity and SCW Synthesis in Populus trichocarpa
Source: Plants (Basel). 2025 Dec 25;15(1):68. doi: 10.3390/plants15010068 (PMC12787359; doi:10.3390/plants15010068)
Supplement: Supplementary file 1 [file plants-15-00068-s001.zip › Table S1 and S2.pdf]

Table S1. Primer list.

Primer name	Forward primer sequence (5′→3′)	Reverse primer sequence (5′→3′)
qBTB82	TGCTGGACTCAGATGCCTACA	GGCATATGCTCAGATTCCTG
BTB82CDS	GGATCCATGGACTGCTCAATTTGT	GTCGACAACTCCACTATTTCTTCT
ProBTB82-1	ATCTTAACAACATAAACTTCAA	TTGAAGATTTGCCTTTATTTGTATAAC
ProBTB82-2	GAGCTCATCTTAACAACATAAACTTCAA	TCTAGATTGAAGATTTGCCTTTATTTGTATAAC
PXY	TGGCGAGCTACCATACCTTGA	CGGAGAGCGAGTTGTTTGAGA
WOX4	GAACCCCATCTCCCGTTACCATT	AGAGGTGGAGGGAGAGGAGGATAGT
WOX14	GAGAACTCCTAATGGGCAACAAA	CATTCAAATGACCAGCTCCTACA
CLE41	GATCACACCATTTCTTTCTCTTCC	GGGCTAGTGAGTAAAATAAAAACGA
CLE20	ACTCTTTCACCTCTTCCTCTTGC	GAACCTTTCGCTTCTGTGACTC
IAA9	TACTGAATTGCGGCTTGGG	CTCTGAAAACTCATCCATGGCAT
IAA12.1	GTTGGGTCTGGGGCTGAGTCTT	CACAGGTAGAAGGCATAGAGT
IAA12.2	GGAGCTGGGGCTGAGTCTTGG	GCAAGCAGAGGAAATAGAAG
ARF5.1	CCAGCAAGCATCCAAGTGAATT	TGTGACTTTTCATCCCTGATG
ARF5.2	ACCAATCCAATGCCATGA	ATAGGCCAGAGGCTGGCTTG
ARR7	ACCAGATGTTTGGAGGAGGGA	TCTTACTGGCTTCAAGAAAAATTCC
ARR15	CATCTGAGAACATCTTGGCTCG	CTGCCCCTTCTTCTAAACACCTAT
HB7	GCAGAAATGTTGCCTAGCGG	GCCGCCATTGTTGTCTTCTG
VCM1	AAGGCAGGAGTTGAACGTGTCC	CCACCATGCAGCTCATGCAATC
VCM2	ACTGGAGCGACAGTTAAAGACAGG	TCCTCTTGAAGGGCTCTTTGCTC
PIN1	ACCAGCAGTTCAAGAACCAG	AAAGTGGCACCATTGCAGTC
PIN3	GCAAAATAGCAAGGCAAACC	AGTTTTGGTTTCCCCGTTTT
PIN5	CGAAGGTTCAGACGTAGCTAAATATT	AACAAACAAAGCGGTTTATGG
PIN6	ATCACCGACGTTGAAGAACA	CGGGTTAGCATGCTGTAATG
BIL1	GCAGGACAGAAGATATAAGAAT	GTTTCACATAAATAAGCGGC
VCS2	AAGAAGAACGGAAGAACCTTCAAG	AAGAAGAACGGAAGAACCTTCAAG
VCS2h	AGTTCATGGCAAGTGGTGAAGAAGGC	GTGGAAACAGAGGTGGCATGTGATTG
JAZ5	ACCAGCGAATCAACCCCGAAT	TGTTTTGGCACGAAATGTGACCT
GRF15	TGGTTGTTCTGTTGTCTT	CGAAGAAGATCCAAGAAGA
MYB31	CCAAGTCCAATATTCTACTG	GTGATGGTTGAAGCACTAAC
C3H17	GGGTGTGATGAGGATGATGATTGGG	GGAGGAAGAGGATTTGATGTGGGCC
DET2	ATGGCCCTATTAGATCAGAGC	GGCTTTCCATGAGGATCCAA
PAL1	CCATCCAGGTCAAATTGAGGCTGCT	ACTTCTTAGCTGCCTTCATGTAAGCT
PAL2	CCTAGAAGCCATCACCAAGTTGCTC	GTTTCTCCATTGGGTCCCACG
PAL3	CATCCAGGTCAAATTGAGGCTGCA	ACTTCTTAGCTGCCTTCATGTAAGC
PAL4	GAGATGCTGGAAGCTATCACCAAAT	GGCTCTCCATTGGGTCCAACT
PAL5	GAGATGCTGGAAGCTATCACCAAGC	GGCTCTCCATTGGGTCCAACT
C4H1	AGTGCGCCATAGACCATATCCTC	ATTGCAGCGACGTTGATGTTCTCA

C4H2	GAAATGTGCAATTGATCATATTTG	ATTGCAGCAACATTGATGTTCTCC
4CL3	ACTAGCCCATCCAGAGATATCCGA	TCATCTTCGGTGGCCTGAGACTTT
4CL5	GTGATCATGCTCATCCTGCCAAGT	TTGGCAGCAGTAGTAATGGCACCT
HCT1	ATCAGCATGTAAGGCACGCGG	TGCCAAAGTAACCAGGTGGAAGCGT
HCT6	AGATCAACATGCAAAGCACGTGA	GCCAAAGTAACCAGGAGGGAGTTG
CCoAOMT 1	CAGTAATTCAGAAAGCTGGTGTTGC	GCATCCACAAAGATGAAATCAAAAC
CCoAOMT 2	CCTTCCAACGCCAGGAAAGAGAGTA	GTGGCCAACTTCTTGATGCCTTCCG
CCoAOMT 3	TGATCGAACCTGCTGTAAAGGGCA	TGCACACAGCAACCATAGAGGACA
CCR2	CGGTGATTCAGAAAGCTGGTCTGGA	GCATCCACAAAGATGAAGTCATAAG
F5H1	AATCCAATATAGGCAAGCCTGTGAACG	ATTTTTGGCCCCAAAAGCTGCTCTA
F5H2	AAGCCAATATAGGCAAGCCTGTGAATC	ATTTTTAGCCCCGAAAGCTGCTCTG
COMT2	TCTTGAAGAATTGCTATGACGCCT	GAATGCACTCAACAAGTATCACCTTG
CAD1	GGCAAGCTGATCTTGATGGGTGTT	TCCCGGTGATTGACTTTCTCCCAA
CSE1	GATACTGGCTGGCTGTTTCA	CATGTAGCAGCGTAAACCGT
CSE2	GGTGACATGGACAAGATTGC	AAGGCTGGTAAGCCCTTGTA
CesA4	GACGATGCCGAGTTTGGAGAGC	CAACTGTGCAAAAGTGGGATAACCT
CesA7A	GGAATTCAGGGTCCGGTGTACG	AATAGACCTCCATCCACGGCAA
CesA7B	AAGGCAAGCTTTGTATGGCTATGAC	AATAGACCTCCAGCCACGACAA
CesA8A	TTGTTGGTGTTGTTGCGGGATT	CATCTCGCAGTTCATGTAACTCAACTACT
CesA8B	CTGGCATCGATACGAACTTCAC	TCTTGAGAGAAACTACAACGAGGA
CCoAOMT 1	CAGTAATTCAGAAAGCTGGTGTTGC	GCATCCACAAAGATGAAATCAAAAC
XCP1	TGTTGAGCTATTCGAATCATGG	GATCAGCTGTTGTTCTGACAA
XCP2	GAAGACTTGACGTCTAGGGATA	ATGCTCGAGACATCCTTGTATGT
XCP3	ACCCCTGAAGACTTGACGTCCG	ACATGACCCCTGGTTCTTGACA
IRX7	ACGGACGTTTGACCCATACGAA	TCCGGCGAGATGTAAGGTGGTA
IRX8A	AGCTAGAAGGGTTTCTTATCGA	TGATGCTACATGCCTGTACAG
IRX8B	CACCAAGTTTGAGGCATGTAA	CCTCCAATGTCTGAGGAATATC
IRX9A	AGTTAGCGCCAAGACGACTGGC	GTTTGCTGAAAGTAATGCCATT
IRX9B	ATTAAGCTCCAAGGAATCTGAA	TGCTCAATGTGCCTTAAAGCAA

Table S2. The gene IDs and gene names of the 103 BTB genes in *P. trichocarpa*.

Gene id	Gene name
Potri.001G086100	*PtrBTB1*
Potri.001G096100	*PtrBTB2*
Potri.001G100900	*PtrBTB3*
Potri.001G102300	*PtrBTB4*
Potri.001G132300	*PtrBTB5*
Potri.001G184200	*PtrBTB6*
Potri.001G233900	*PtrBTB7*
Potri.001G255950	*PtrBTB8*
Potri.001G280100	*PtrBTB9*
Potri.001G295600	*PtrBTB10*
Potri.001G357100	*PtrBTB11*
Potri.001G457550	*PtrBTB12*
Potri.001G468700	*PtrBTB13*
Potri.002G010100	*PtrBTB14*
Potri.002G018700	*PtrBTB15*
Potri.002G048300	*PtrBTB16*
Potri.002G052700	*PtrBTB17*
Potri.002G056500	*PtrBTB18*
Potri.002G077000	*PtrBTB19*
Potri.002G082800	*PtrBTB20*
Potri.002G166700	*PtrBTB21*
Potri.002G209700	*PtrBTB22*
Potri.002G242300	*PtrBTB23*
Potri.003G129300	*PtrBTB24*
Potri.003G131000	*PtrBTB25*
Potri.003G135300	*PtrBTB26*
Potri.004G175900	*PtrBTB27*
Potri.004G189800	*PtrBTB28*
Potri.004G194200	*PtrBTB29*
Potri.005G058700	*PtrBTB30*
Potri.005G075400	*PtrBTB31*
Potri.005G129700	*PtrBTB32*
Potri.005G130700	*PtrBTB33*
Potri.005G146400	*PtrBTB34*
Potri.005G149400	*PtrBTB35*
Potri.005G178400	*PtrBTB36*
Potri.005G183600	*PtrBTB37*
Potri.005G206100	*PtrBTB38*
Potri.005G210000	*PtrBTB39*
Potri.005G214400	*PtrBTB40*
Potri.005G251300	*PtrBTB41*
Potri.006G003000	*PtrBTB42*

Potri.006G043400	*PtrBTB43*
Potri.006G103400	*PtrBTB44*
Potri.006G148100	*PtrBTB45*
Potri.006G185300	*PtrBTB46*
Potri.006G202900	*PtrBTB47*
Potri.006G264300	*PtrBTB48*
Potri.007G032800	*PtrBTB49*
Potri.007G033900	*PtrBTB50*
Potri.007G053200	*PtrBTB51*
Potri.007G055100	*PtrBTB52*
Potri.007G093000	*PtrBTB53*
Potri.007G109300	*PtrBTB54*
Potri.007G112600	*PtrBTB55*
Potri.007G118800	*PtrBTB56*
Potri.007G140400	*PtrBTB57*
Potri.008G038600	*PtrBTB58*
Potri.008G059600	*PtrBTB59*
Potri.008G085500	*PtrBTB60*
Potri.008G150100	*PtrBTB61*
Potri.008G186100	*PtrBTB62*
Potri.008G200700	*PtrBTB63*
Potri.009G037800	*PtrBTB64*
Potri.009G075300	*PtrBTB65*
Potri.009G089500	*PtrBTB66*
Potri.009G150500	*PtrBTB67*
Potri.009G156500	*PtrBTB68*
Potri.010G014000	*PtrBTB69*
Potri.010G029500	*PtrBTB70*
Potri.010G046800	*PtrBTB71*
Potri.010G090900	*PtrBTB72*
Potri.010G170900	*PtrBTB73*
Potri.010G199200	*PtrBTB74*
Potri.010G223600	*PtrBTB75*
Potri.011G091100	*PtrBTB76*
Potri.012G091400	*PtrBTB77*
Potri.012G118300	*PtrBTB78*
Potri.012G118500	*PtrBTB79*
Potri.013G024400	*PtrBTB80*
Potri.013G062500	*PtrBTB81*
Potri.013G083800	*PtrBTB82*
Potri.013G159000	*PtrBTB83*
Potri.014G093700	*PtrBTB84*
Potri.014G133500	*PtrBTB85*
Potri.014G163200	*PtrBTB86*

Potri.014G164000	*PtrBTB87*
Potri.015G087700	*PtrBTB88*
Potri.015G117200	*PtrBTB89*
Potri.016G003700	*PtrBTB90*
Potri.016G040500	*PtrBTB91*
Potri.016G090400	*PtrBTB92*
Potri.016G112900	*PtrBTB93*
Potri.016G123800	*PtrBTB94*
Potri.016G139900	*PtrBTB95*
Potri.017G009700	*PtrBTB96*
Potri.017G041600	*PtrBTB97*
Potri.017G048200	*PtrBTB98*
Potri.018G018600	*PtrBTB99*
Potri.018G107600	*PtrBTB100*
Potri.019G038400	*PtrBTB101*
Potri.019G039500	*PtrBTB102*
Potri.019G131600	*PtrBTB103*
